# Brain activity during driving with distraction: an immersive fMRI study

**DOI:** 10.3389/fnhum.2013.00053

**Published:** 2013-02-28

**Authors:** Tom A. Schweizer, Karen Kan, Yuwen Hung, Fred Tam, Gary Naglie, Simon J. Graham

**Affiliations:** ^1^Keenan Research Centre of the Li Ka Shing Knowledge Institute, St. Michael's HospitalToronto, ON, Canada; ^2^Department of Surgery, Faculty of Medicine, Division of Neurosurgery, University of TorontoToronto, ON, Canada; ^3^Institute of Biomaterials and Biomedical Engineering, University of TorontoToronto, ON, Canada; ^4^Department of Physical Sciences, Sunnybrook Research InstituteToronto, ON, Canada; ^5^Department of Medicine, Baycrest Geriatric Health Care CentreToronto, ON, Canada; ^6^Rotman Research Institute, BaycrestToronto, ON, Canada; ^7^Toronto Rehabilitation Research Institute, University Health NetworkToronto, ON, Canada; ^8^Department of Medicine and Institute of Health Policy, Management and Evaluation, University of TorontoToronto, ON, Canada; ^9^Department of Medical Biophysics, Faculty of Medicine, University of TorontoToronto, ON, Canada

**Keywords:** driving, driving distractions, neural correlates of driving, driving complexity, fMRI

## Abstract

**Introduction:** Non-invasive measurements of brain activity have an important role to play in understanding driving ability. The current study aimed to identify the neural underpinnings of human driving behavior by visualizing the areas of the brain involved in driving under different levels of demand, such as driving while distracted or making left turns at busy intersections.

**Materials and Methods:** To capture brain activity during driving, we placed a driving simulator with a fully functional steering wheel and pedals in a 3.0 Tesla functional magnetic resonance imaging (fMRI) system. To identify the brain areas involved while performing different real-world driving maneuvers, participants completed tasks ranging from simple (right turns) to more complex (left turns at busy intersections). To assess the effects of driving while distracted, participants were asked to perform an auditory task while driving analogous to speaking on a hands-free device and driving.

**Results:** A widely distributed brain network was identified, especially when making left turns at busy intersections compared to more simple driving tasks. During distracted driving, brain activation shifted dramatically from the posterior, visual and spatial areas to the prefrontal cortex.

**Conclusions:** Our findings suggest that the distracted brain sacrificed areas in the posterior brain important for visual attention and alertness to recruit enough brain resources to perform a secondary, cognitive task. The present findings offer important new insights into the scientific understanding of the neuro-cognitive mechanisms of driving behavior and lay down an important foundation for future clinical research.

## Introduction

Driving is an essential daily activity for many people, providing mobility, independence, and sometimes a source of livelihood. Previous research has reported factors that increase the risk of vehicle crashes and driving errors including fatigue and sleepiness after extended driving (Sagaspe et al., 2008) and alcohol consumption (De Boni et al., 2012). Research has also reported on neurological factors that impact driving ability, especially in individuals with cognitive impairments (Rapoport et al., 2007; Carr and Ott, 2010; MacDonald and Hébert, 2010; Nelson, 2010), that increase the risk of vehicle crashes (Rizzo, 2011). Health professionals have the responsibility to identify individuals with medical conditions that may detract from safe driving ability (Carr et al., 2006). However, to date there has been no consensus on the assessment of fitness to drive and it remains a significant challenge for clinicians to evaluate a patient's driving capacity (Eby and Molnar, 2010). Some individuals may be able to drive in simple, well-practiced circumstances, but may be incapable of driving safely when circumstances become more demanding or novel. There is very limited knowledge about how different levels of driving behavior are supported by normally functioning brains, and how different areas of the brain interact when performing various driving tasks.

Safe driving requires the ability to concentrate, to divide attention between multiple sensory events across visual and auditory modalities, and to make fast cognitive decisions in a complex and rapidly changing environment. The present study applied virtual reality (VR) technology in a functional magnetic resonance imaging (fMRI) system to investigate how brain responses of healthy adults change across various driving scenarios. Using this novel setup allowed us to obtain a real-time, spatiotemporal profile of brain activity while driving in a safe environment. Previous fMRI driving research has identified brain areas responsive to different aspects of driving (Carvalho et al., 2006; Spiers and Maguire, 2007), including maintaining driving speed (Peres et al., 2000; Calhoun et al., 2002) and responding to uncertainties during driving (Callan et al., 2009). These studies commonly identified brain regions involving the motor, parietal, occipital, and cerebellar cortices responsible for various driving maneuvers (Walter et al., 2001; Calhoun et al., 2002; Uchiyama et al., 2003; Graydon et al., 2004; Calhoun and Pearlson, 2012) such as turning, reversing, and stopping (Spiers and Maguire, 2007) all of which require visual-spatial and visual-motor processes. Specific tasks such as monitoring other cars, processing traffic rules, and action planning, recruit the pre-supplementary motor area, the superior parietal and lateral occipital cortices, as well as the cerebellum (Spiers and Maguire, 2007). These additional activations in posterior brain regions have been attributed to the increased demands that driving places on vision and motor skills, as well as visuo-motor and visuo-spatial integration. Previous research examining driving performance with distractions has reported an associated reduction in brain resources (Just et al., 2008). However, existing studies have provided limited data related to real-world driving behaviors, such as driving with distraction.

Cognitive psychologists have proposed that the posterior attention system engages brain areas such as the occipital-parietal and posterior cingulate regions that are critical to visual-spatial orientation and integration functions. In contrast, the anterior attention system serves a higher-level attention function, engaging anterior portions of the frontal lobes such as the prefrontal and anterior cingulate regions, which are responsible for executive attentional control in more complex cognitive tasks associated with problem-solving and decision-making, especially during multi-tasking (Posner and Dehaene, 1994; Posner, 2012). Studies have used electroencephalography (EEG) techniques to estimate possible intracerebral sources associated with driving-related processing: deactivation of signals during fast driving related to a drop of cognitive control has been reported (Jancke et al., 2008); and visual event-related potentials (ERP) components were diminished and the attentional selection of target stimuli were less efficient during concurrent auditory dual-tasks (Gherri and Eimer, 2011). Magnetoencephalography (MEG) studies with driving simulators have reported multiple brain sources, including visual, parietal, and frontal areas that are engaged during visual attention to driving-related signals (traffic lights and direction signs), with increased attention demand in a dual-task audio condition modifying the neural processing of visual signals (Fort et al., 2010). Functional Near Infrared Spectroscopy (FNIRS) has also been used to explore the neural underpinnings of driving behavior. Recent studies have suggested that the frontal lobes are sensitive to the type of driving task, with greater frontal lobe activity observed for externally directed driving behavior (following audio driving instructions) compared to internally-driven driving behavior (based on memory) (Liu et al., 2012). FNIRS data also suggest that parietal and occipital brain areas are responsible for spatial attention in the perception of VR space (Seraglia et al., 2011; also see the review of Calhoun and Pearlson, 2012).

The current study focused on identifying the underlying neural networks subserving different driving behaviors including distracted driving. Our immersive and novel VR setup (Kan et al., 2013) allowed us to investigate how the brains of healthy young adult drivers respond during various simulated driving conditions ranging in levels of complexity. This goal was achieved by using virtual driving technology combined with advanced neuroimaging techniques. Driving tasks were designed to require increasing levels of attentional processing demands and visual complexity. For example, the tasks included making turns at intersections with and without oncoming traffic, or driving under auditory distractions that mimic driving while talking on a hands-free cell phone or engaging in a conversation with a passenger. These simulated driving conditions allowed us to tap into the same neural processes associated with a more complete range of real-world driving behaviors. The cognitive manipulation during driving in the current study can be helpful in studying the underlying neural substrates for various driving behaviors, such as distracted driving. The knowledge of these neural substrates ultimately can help health professionals to more effectively assess driving competence in individuals with brain dysfunctions (e.g., using office-based, cost-effective tests), and will eventually contribute to the design of potential cognitive rehabilitation strategies.

We hypothesized that complex simulated driving conditions (such as making turns, particularly left turns at busy intersections while encountering oncoming traffic) would involve posterior brain activations including motor and occipital-parietal regions for visual-spatial and visual-motor integration. On the other hand, prefrontal activation would be involved in a distracted condition consisting of performing a secondary cognitive task during simulated driving, related to the executive function demands when attentional resources are divided across multiple tasks.

## Materials and methods

### Ethics statement

Ethical approval for the study was obtained on July 18th 2010, by the Research Ethics Board at Baycrest Hospital in Toronto, Canada. All participants provided written informed consent prior to participating in the study.

### Participants

Participants were recruited through the university network via advertisement and emails. All participants were right-handed with normal or corrected vision. Participants without a valid driver's license, with a history of psychological or neurological illness, or with fMRI contraindications (such as having claustrophobia or ferromagnetic implants) were excluded. Sixteen participants (7 females and 9 males) between the ages of 20 and 30 years (Mean = 25.8, *SD* = 1.5) who were actively driving and with mean driving experience of 7.4 years (*SD* = 2.5) were studied.

### Driving simulation

We applied a novel approach using an immersive VR environment in a 3.0 Tesla MRI system to capture brain activity with high ecological validity (Kan et al., 2013). We applied driving hardware (steering wheel and foot pedals) custom built for compatibility with fMRI. The driving scenario was designed using STISIM Drive software (Systems Technology Inc., Hawthorne, CA). Participants viewed the simulation through a mirror attached to the head coil (see “Neuroimaging” section), which oriented on a projection screen illuminated by an LCD projector system projecting through a waveguide in the radiofrequency shield of the MRI room; participants also wore fMRI-compatible headphones (Silent Scan, Avotec, Inc., Stuart, FL) to hear the audio tasks.

### Driving tasks

**Figure 1:**
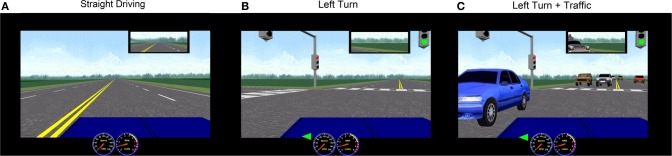
**Representative STISIM screenshots of simulated driving conditions used in the fMRI.** Rural scenery was chosen to minimize the potential confounding variations from using complex visual backgrounds. **(A)** Straight driving; **(B)** Left turn at intersection with no traffic; **(C)** Left turn with oncoming traffic.

**Figure 2:**
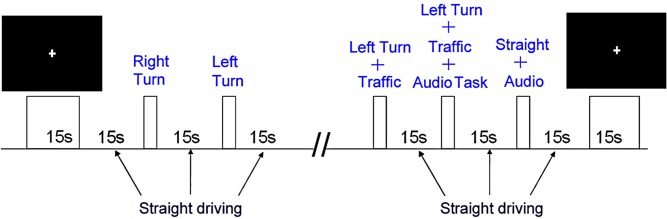
**The experimental task design**.

Prior to fMRI, participants underwent an hour-long training session in an fMRI simulator to practice simulated driving. The tasks included straight driving (“Straight Driving,” Figure 1A), turning at intersections with and without oncoming traffic, or driving while performing audio tasks. Each participant performed six simple right-hand turns (“Right Turn”) and six left-hand turns (“Left-Turn,” Figure 1B) with no traffic. To increase driving complexity, participants encountered six left turns with a stream of oncoming traffic (“Left Turn + Traffic,” Figure 1C), which required participants to decide when to turn safely. In conditions of distracted driving, participants were presented with concurrent audio tasks consisting of general knowledge true or false questions (e.g., “a triangle has four sides”) during straight driving (“Straight + Audio”; six times) as well as in the demanding turns (“Left Turn + Traffic + Audio”; six times). Participants answered the questions by pressing corresponding buttons embedded on the steering wheel (similar to modern vehicle designs for answering hands-free devices or volume controls). The experimental protocol is shown in Figure 2. Straight driving served as the control condition (baseline) interspersed between other specific tasks in pseudo-random order. Each task was introduced by a voice recording (e.g., “At the intersection, turn left”) approximately 5 s prior to the task, similar to navigation instructions with a Global Positioning System. Participants were asked to follow the traffic rules and to drive as close to the posted speed limit as possible. Each driving task was separated by at least 15 s of straight driving (baseline/controls) to maintain separation to limit overlapping of the fMRI hemodynamic response signals.

### Neuroimaging

Participants received motion training to operate the driving controls with minimal head motion and practiced on four training runs that introduced all driving conditions. During testing, participants were placed in the MRI system with the driving hardware in a comfortable position. A high-resolution anatomical scan was acquired first, followed by three fMRI runs with simulated driving. The driving scenarios were triggered synchronously with the fMRI time series data collection. Each run was approximately 9–9.5 min. Images were acquired using a research-dedicated whole body 3.0 Tesla MRI system (Magnetom TIM Trio software version b15, Siemens, Erlangen, Germany). The high-resolution anatomical scan was acquired with T1-weighted, 3D magnetization prepared rapid gradient echo imaging (MPRAGE; echo time (*TE*) = 2.63 ms, 160 slices, thickness = 1.0 mm, gap = 0 mm, field of view (*FOV*) = 256 × 192 mm, matrix = 256 × 192, yielding 1 × 1 × 1 mm voxels). Functional MRI was undertaken using T2^*^-weighted echo planar imaging (EPI; repetition time (*TR*) = 2 s, *TE* = 30 ms, flip angle = 70 degrees, 32 slices, thickness = 4.5 mm, gap = 0 mm, *FOV* = 200 × 200 mm, matrix = 64 × 64, yielding 3.13 × 3.13 × 4.5 mm voxels).

### Data analysis

The first 10 s of scanning for each driving run was discarded to allow for equilibration effects. Using AFNI freeware (Cox, 1996), time series data were corrected for physiological movement due to respiration, corrected for slice timing effects, and co-registered to the 16th time point of the first run. The dataset was spatially smoothed using a 5 mm full width at half maximum (FWHM) Gaussian kernel and normalized by the run-wise mean of each voxel. Statistical brain activation maps were calculated using a General Linear Model (GLM) by convolving a stimulus-timing file with a variable-shape hemodynamic response function (HRF) with seven regressors (for an expected HRF of 14 s, one regressor was used per TR). Estimated head motion parameters, in six degrees of freedom as determined from the co-registration procedure indicated above, were included as nuisance regressors as well as a 4th order polynomial for baseline detrending. The resulting GLM parameter estimates were summed, transformed into Talairach brain atlas space (Talairach and Tournoux, 1988), spatially smoothed using a 6 mm FWHM Gaussian kernel, and the maps for all 16 subjects were entered into a within-subjects, random effects ANOVA. Finally, t-statistic maps for all conditions were thresholded using a false discovery rate method (Genovese et al., 2002) at a level of *q* = 0.05, to correct for multiple statistical comparisons. For interpretation, the final group activation maps were overlaid on the average of all 16 anatomical images, which were also transformed into Talairach brain atlas space. To disentangle the visuo-motor and higher cognitive functions involved in driving, straight driving was used as the control condition in comparison with all other driving conditions. The statistical comparisons of interest across group activation maps were, therefore: distracted straight driving vs. control; right turns vs. control; left turns vs. control; complex turns vs. control; and distracted complex turns vs. control.

## Results

### Brain activations

**Figure 3:**
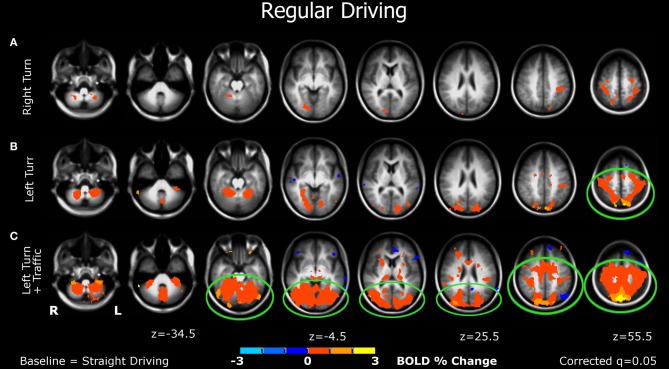
**Brain activations from the bottom to the top of the brain (left to right figures) of participants when performing various simulated driving conditions. (A)** The right-turn condition showed minimal activation in the brain; **(B)** Left-turn showed more activation in the posterior brain regions; **(C)** The left-turns with oncoming traffic generated larger significant activations of multiple bilateral regions in the mid-posterior brain areas. See details in the “Results” section.

**Figure 4:**
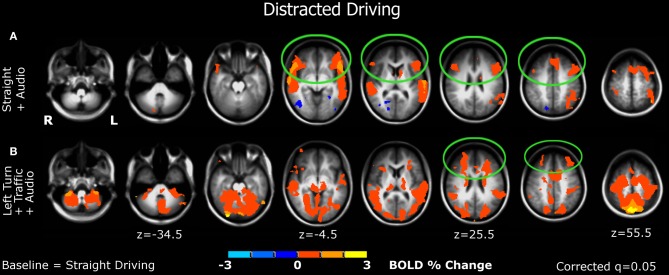
**Brain activations associated with distracted driving. (A)** Straight driving with a cognitive-distraction, audio task. **(B)** The demanding, left-turn condition with oncoming traffic plus the cognitive distraction.

Significant brain activations are summarized below, reported relative to the straight driving control condition. Brain activation images for each task are shown in Figures 3 and 4. Peak locations of brain activity are reported in Table 1. All reported results were in contrast with the baseline (control condition: straight driving). Behavioral results can be seen in Appendix.

**Table 1:** **Peak locations (Tailarach coordinates) of brain activations during each driving task**.

**Peak activations**	***L***	***P***	***I***	**TT Atlas**
**RIGHT TURNS**				
	−24	−60	−62	L superior parietal lobule, BA7
	−14	−78	44	L precuneus, BA7
	34	−40	64	R postcentral gyrus, BA5
	20	−66	58	R superior parietal lobule, BA7
	18	−72	−8	R lingual gyrus, BA18
**LEFT TURNS**				
	−44	−30	60	L postcentral gyrus, BA1/2
	−6	−62	64	L precuneus, BA7
	−4	−86	38	L cuneus, BA7
	12	−76	56	R precuneus, BA7
	34	−14	66	R precentral gyrus, BA6
	38	−34	64	R postcentral gyrus, BA1/2
	58	−44	−34	R cerebellum (Crus 1)
**LEFT TURNS + TRAFFIC**				
	−30	−28	68	L precentral gyrus, BA4
	−28	−4	64	L superior frontal gyrus, BA6
	−8	−74	56	L precuneus, BA7
	−2	−84	−18	L lingual gyrus, BA18
	−20	−72	−14	L fusiform gyrus, BA19
	−32	−36	−50	L cerebellum (VIII)
	32	−12	66	R precentral gyrus, BA6
	36	−34	66	R postcentral gyrus, Ba1/2
	34	−84	26	R superior occipital gyrus, BA19
	4	−96	18	R cuneus, BA18
	10	−76	54	R precuneus, BA18
	26	−92	−18	R fusiform gyrus, BA18
	30	−36	−50	R cerebellum (VIII)
**LEFT TURNS + TRAFFIC + AUDIO**				
	−2	−98	−2	L cuneus, BA18
	−6	−72	58	L precuneus, BA7
	−4	−84	−20	L lingual gyrus, BA18
	−46	−70	−16	L fusiform gyrus, BA19
	−2	−34	74	L paracentral lobule
	−32	−36	−52	L cerebellum (VIII)
	68	−14	0	R superior temporal gyrus, BA22
	34	−12	66	R precentral gyrus, BA6
	36	−34	66	R postcentral gyrus, BA1/2
	2	−96	20	R cuneus, BA18
	24	−88	−16	R fusiform gyrus, BA18
	4	−74	54	R precuneus, BA7
	30	−38	−52	R cerebellum (VIII)
**STRAIGHT DRIVING + AUDIO**				
	−66	−28	2	L middle temporal gyrus, BA21
	−64	−48	−10	L inferior temporal gyrus, BA37
	−64	−8	6	L superior temporal gyrus, BA22
	−54	20	−6	L inferior frontal gyrus, BA47
	−46	−56	52	L inferior parietal lobule, BA40
	−32	−76	48	L superior parietal lobule, BA47
	54	22	−4	R inferior frontal gyrus, BA47
	68	−14	2	R superior temporal gyrus, BA22

BA, Brodmann area.

#### Regular Driving

***Right turn (Figure 3A).*** Minimal significant activation was observed in this simple task, including the somatosensory association (postcentral gyrus), parietal (including precuneus), and visual cortices (lingual gyrus).

***Left turn (Figure 3B).*** More activation was detected in this condition, which was observed in the premotor cortex, somatosensory area, visual and parietal cortices, as well as the cerebellum.

***Left turn + traffic (Figure 3C).*** This condition showed larger significant activations of multiple bilateral regions in the mid-posterior brain, including motor and premotor areas, visual, parietal, and somatosensory regions, and the cerebellum.

#### Distracted driving

***Straight + audio (Figure 4A).*** Significant activations were found in the ventrolateral prefrontal cortex (vlPFC) bilaterally, in addition to auditory cortex. Other activations were detected in the parietal lobes. Decreased activation in occipital-visual regions was observed.

***Left turn + traffic + audio (Figure 4B).*** In addition to auditory, motor, somatosensory, visual, parietal, and cerebellar regions, significant additional activations were detected in anterior brain areas bilaterally, mainly in the dorsolateral prefrontal cortex (dlPFC) and the frontal polar region.

### Behavioral results

Driving performance showed an effect of speed differences among undistracted driving conditions (*p* < 0.05; Repeated Measure ANOVA; Table A1); *post-hoc* comparisons showed that the mean speed in left-turn-traffic was the slowest (29.4 km/hr, *SD* = 4.3 km/hr) compared to left turns (26.8 km/hr, *SD* = 5.2 km/hr) and right turns (24.0 km/hr, *SD* = 3.3 km/hr). Left turn speed was significantly slower than right turn speed, which was the fastest among all conditions significantly (*p* < 0.05, Least Significant Difference *post-hoc* tests; Table A2). The speed in the distracted left-turn-traffic condition was not significantly different from the left turn and left-turn-traffic conditions and was only slower than right turns (*p* < 0.05). Average speed during straight driving (58.57 km/hr, *SD* = 3.36 km/hr) was not significantly different from that of distracted straight driving (58.69 km/hr, *SD* = 2.34 km/hr) (Table A3). Lane position during straight driving (2.35 km/hr, *SD* = 0.31 km/hr) was not significantly different from that of distracted straight driving (2.51, *SD* = 0.42 km/hr) (Table A3). Average response accuracy to the audio distraction task was 87% (range = 50–100%, above the chance level).

## Discussion

The current study extends previous research by using an immersive fMRI-compatible driving simulator to examine how the human brain responds to various driving conditions, and by characterizing the effects of cognitive distraction on driving. First, we observed that the patterns of brain activation depend on the type of simulated driving task. Performing right turns, the simplest task, generated minimal activation relative to the control condition (Figure 3A). Making left turns, without oncoming traffic, the participants showed activations in the posterior brain, including visual-parietal and motor areas (Figure 3B), suggesting that cognitive resources involving visuospatial and motor coordination are required for making left turns. Performing the more demanding left turns at busy intersections, where in the real world most serious crashes occur (National Highway Traffic Safety Administration, 2009; Choi, 2010), produced larger activations in the posterior network, along with additional activation of the cingulate cortex, an area important for cognitive-response selection and alertness (Vogt et al., 2004) (Figure 3C).

Second, a significant shift in activation from the posterior to the anterior brain was observed when driving became distracted. Compared to straight driving, auditory distraction during straight driving significantly activated not only auditory areas but also the prefrontal cortices (mainly in the ventral lateral prefrontal cortex regions; Figure 4A), while decreased activation in posterior brain regions was evident. These findings support the study hypothesis in that undistracted driving, even in attentionally demanding conditions, engaged the posterior brain system, while driving under cognitive distractions activated the anterior brain system. Consistently, when the more challenging maneuver (turning left at a busy intersection) was performed under cognitive distraction, the anterior network was additionally engaged (Figure 4B), predominantly in the dorsolateral prefrontal cortex/frontal pole. These regions are associated with executive functions including attention and working memory processes, and processing thoughts and decision-making critical for multitasking (Christoff and Gabrieli, 2000).

To substantiate the observed shift from occipital to frontal brain activations, particularly in the prefrontal areas when comparing the left-turn-traffic condition to the left-turn-traffic plus cognitive distraction, we extracted mean BOLD percentage change values for each subject from the activated occipital and prefrontal regions of interest, and conducted *post-hoc* tests of the differences between task conditions using Matlab (Mathworks Inc., Natick, MA). Results confirmed a significant decrease in the mean occipital activation [from 0.59 to 0.36%, *p* = 0.001, paired samples *t*_(15)_ = 4.01] and an increase in the mean prefrontal activation [from 0.11 to 0.39%, *p* = 0.016, paired samples *t*_(15)_ = −2.72].

This anterior-vs.-posterior shift in BOLD signals reflects changing reactions of the brain, and highlights the effect of distracted driving and the role of the anterior frontal region, an area that has been associated with impulsiveness (e.g., Beeli et al., 2008) critical to driving. The pattern of increased frontal activation accompanied by cognitive distraction has been previously observed in participants performing a visual event detection task while passively watching a driving video under auditory distraction (Hsieh et al., 2009), as well as during performing divided attention, dual-tasks involving both visual and auditory modalities compared to performing single-modality tasks (Schubert and Szameitat, 2003; Johnson and Zatorre, 2006). One important caveat is that the prefrontal activity observed when the participants were performing simultaneous driving and auditory tasks may not be entirely associated with the distraction, but may be at least partly related to auditory attention needed for the secondary task. Future studies will be required to analyze this issue in more detail.

Supporting the findings and interpretation of the present work, an observation of decreased activation in parietal-visual areas and impaired driving performance in a dual-task driving condition involving concurrent language comprehension has also been previously reported (Just et al., 2008). We provided data within a single study demonstrating a response shift between these brain areas from the posterior to the anterior networks related to driving distraction. This interplay between anterior and posterior brain regions is possibly related to a competition for limited resources and attentional reallocation between the anterior, executive attention in multitasking and the posterior, visual-response attention system (Rees et al., 1997; Wickens, 2008). The brain may face a “bottleneck” when multiple tasks simultaneously compete for shared and limited resources, constraining available resources for individual tasks (Just et al., 2001; Dux et al., 2006). This view suggests that with cognitive distraction during driving, to support mental processing in the anterior brain, resources of the posterior brain important for visual alertness and visual attention were sacrificed.

The current finding has important implications regarding distracted driving. While changes in driving performance observed in the undistracted conditions (slowing down from right turns to left turns and traffic) were parallel to the results of brain activations in the posterior brain (increases in activated areas), brain activity shifted to the anterior network when there was no behavioral change from the undistracted to the distracted condition. Eye-tracking studies have shown that hands-free conversations using cell phones impair attention to visual inputs (Strayer et al., 2003). These distracted drivers experience “inattention blindness”: their field of view narrows (Maples et al., 2008), and they tend to “look at” but not “see” the information in their driving environment (Strayer, 2007), and miss visual cues important for safe driving (Jacobson and Gostin, 2010). As a result, epidemiological findings of real-world collisions show that drivers using hands-free phones are just as likely to experience vehicle crashes as those using hand-held devices (Redelmeier and Tibshirani, 1997; McEvoy et al., 2005). The present study provides neuroimaging evidence supporting previous behavioral observations suggesting that multitasking while driving may potentially compromise visual attention and alertness due to a reduction in brain activation supporting critical visual processing areas, even without significant behavioral changes. Therefore the dangers introduced by distracted driving should be regarded in terms of increased cognitive distractions (i.e., using hands-free cell phones) rather than motor distractions (i.e., physically holding a device) (Strayer and Johnston, 2001).

Previous studies have reported measures that may be able to predict those who passed on-road assessments from those who failed (Baldock et al., 2006). These tests include, for example, The Trail Making Test-B (Richardson and Marottoli, 2003; Staplin et al., 2003; Whelihan et al., 2005), Ergovision Movement Perception Test (De Raedt and Ponjaert-Kristoffersen, 2000), UFOV (De Raedt and Ponjaert-Kristoffersen, 2000), Complex Reaction Time Task (De Raedt and Ponjaert-Kristoffersen, 2000), Paper Folding Task (De Raedt and Ponjaert-Kristoffersen, 2000), Dot Counting (De Raedt and Ponjaert-Kristoffersen, 2000), WMS Visual Reproduction (Richardson and Marottoli, 2003), and Computerized Visual Attention Test Single Task (Mathias and Lucas, 2009). It is clinically important to understand the specific cognitive functions required in different driving circumstances in order to identify “borderline” drivers or those with “restricted” capacities who may be at risk in certain driving conditions. Recent evidence shows that smaller gray matter volume in frontal brain regions was associated with lower executive function capacity and a proclivity to risky driving (Sakai et al., 2012). Given the current finding that prefrontal areas were only significantly activated during the distracted driving conditions, we propose that future assessment should consider using neurocognitive tests that tap into executive, frontal-lobe functions and divided attention for evaluating “fitness for distracted driving.” Based on the current study, it can be inferred that damage to the anterior brain regions may result in specific or restricted impairment (i.e., this type of patient may be unfit to drive safely in distracted circumstances), whereas damage to the posterior brain areas involving the visual, spatial, and motor abilities would significantly compromise general fitness to drive. This implication is also critical for establishing potential restricted driving licensing (Marshall et al., 2002).

In addition, while regular driving (e.g., simple right/left turn without traffic) relies on more learned and automatic processes that activate a driving network in the posterior brain, the mid-cingulate cortex was only differentially activated when the driving conditions became more demanding (left turn with oncoming traffic). This brain region has a major role to play in response selection (Paus et al., 1993; Vogt et al., 2004), attention-for-action (Posner et al., 1988), and working memory (Petit et al., 1998); therefore neurocognitive tests associated with selective attention, response inhibition, and visual-motor abilities can be targeted to evaluate the ability to drive safely in demanding circumstances, even without distraction.

A limitation of the present study is the use of young drivers, which may reduce the generalizability to older populations. Another limitation is that by using simulated driving we were unable to replicate the potential anxiety associated with driving under conditions of increasing complexity, given that there is no real crash risk. Although the use of simulated driving during fMRI may not perfectly generalize to real-world driving, it allows for the investigation of complex driving conditions that are not usually tested during on-road assessments (i.e., left turns during peak traffic or driving while talking on a cell phone). Indeed, previous research suggests that there is a significant correlation between on-road test performance and performance in the driving simulator (Freund et al., 2002). Lundqvist and colleagues have suggested that the predictive ability of driving simulators in assessing actual driving behavior was superior to that of on-road driving tests, partially due to the well-controlled complexity levels in the simulated driving scenarios (Lundqvist et al., 2000). Driving simulators provide a more standardized environment (Michon, 1989; Ranney, 1994) compared to on-road driving tests, offering an ecologically valid and safe way to study human driving behavior in a variety of challenging conditions (Grealy et al., 1999).

## Conclusions

The present study provides new neuroimaging data of the complex brain activity associated with distracted driving and driving under different levels of complexity. We found that brain activations during driving rely on areas important for various cognitive functions including the posterior visual-spatial attentional system vs. the anterior, frontal-lobe functions in multitasking and divided attention. For most people, driving involves highly practiced skills that generally draw on automatic or practiced abilities relying on a posterior network, and does not heavily require the anterior frontal system for more effortful mental processing. However, there are potentially many distractions present during driving (Editorial, 2001), including conversations with passengers, or unpredictable events that increase the risk of crashes. The current findings may be applied to future research on clinical populations with various brain disorders (e.g., Ott et al., 2000; Bedard et al., 2011) to determine how brain damage affects the ability to adapt to daily driving tasks. The shift in brain activation indicates that the assessment of fitness to drive should consider different levels of driving demands and more selective evaluations of driving ability (e.g., in different traffic conditions, or driving while conversing with the examiner). As the brain has limited cognitive resources, this fundamental constraint limits the capacity during driving to carrying out any other cognitive operations such as language comprehension (Newman et al., 2007). Lastly, automobile manufacturers also have a responsibility to improve safety by refraining from installing various communication devices in vehicles, or by installing deactivation systems if drivers attempt to use the devices while the car is in motion (Jacobson and Gostin, 2010). More research is needed to determine if intervention programs (e.g., Devos et al., 2009) that apply simulator protocols with attention training can improve fitness to drive, for example, in improving left turns at busy intersections, or reducing vehicle collision risks for certain brain damaged populations.

### Conflict of interest statement

The authors declare that the research was conducted in the absence of any commercial or financial relationships that could be construed as a potential conflict of interest.

## Appendix

### General linear model: repeated measure ANOVA

Table A1 **ANOVA results showed a significant effect on speed across four driving conditions**.

**Table:**

**Within-subjects factors**	
**Speed condition**	**Dependent variable**
1	Right turn speed
2	Left turn speed
3	Left turn traffic speed
4	Left turn traffic distraction speed

**Table:**

**Descriptive statistics**			
	**Mean**	**Std. deviation**	***N***
Right turn speed	24.0366	3.32044	16
Left turn speed	26.7939	5.16746	16
Left turn traffic speed	29.3542	4.26414	16
Left turn traffic distraction speed	28.9814	3.75817	16

**Table:**

	**Tests of within-subjects effects**			
**MEASURE: MEASURE_1**			
**Source**	**Type III sum of squares**	***df***	**Mean square**
**SPEED CONDITION**			
Sphericity assumed	287.234	3	95.745
Greenhouse-geisser	287.234	2.579	111.382
Huynh–Feldt	287.234	3.000	95.745
Lower-bound	287.234	1.000	287.234
**ERROR (SPEED CONDITION)**			
Sphericity assumed	324.448	45	7.210
Greenhouse-geisser	324.448	38.682	8.387
Huynh–Feldt	324.448	45.000	7.210
Lower-bound	324.448	15.000	21.630

**Table:**

**Tests of within-subjects effects**		
**MEASURE: MEASURE_1**		
**Source**	***F***	**Sig.**
**SPEED CONDITION**		
Sphericity assumed	13.280	0.000
Greenhouse-geisser	13.280	0.000
Huynh–Feldt	13.280	0.000
Lower-bound	13.280	0.002

**Table A2:** ***Post-hoc* tests suggest that speed in the distracted left-turn-traffic condition was significantly slower than both the left and the right turn speed, and the left turn speed was significantly slower than the right turn speed**.

**Pairwise comparisons**				
**MEASURE: MEASURE_1**				
**(I) Speed condition**	**(J) Speed condition**	**^a^**		
		**Mean difference (I-J)**	**Std. error**	**Sig.^a^**
1	2	−2.757^*^	1.078	0.022
	3	−5.318^*^	0.820	0.000
	4	−4.945^*^	0.763	0.000
2	1	2.757^*^	1.078	0.022
	3	−2.560^*^	0.997	0.021
	4	−2.187	1.110	0.068
3	1	5.318^*^	0.820	0.000
	2	2.560^*^	0.997	0.021
	4	0.373	0.873	0.676
4	1	4.945^*^	0.763	0.000
	2	2.187	1.110	0.068
	3	−0.373	0.873	0.676

The right-turn speed was the slowest (p < 0.05, LSD post-hoc tests).

Based on estimated marginal means:

* The mean difference is significant at the 0.05 level.

a Adjustment for multiple comparisons: least significant difference (equivalent to no adjustments).

Table A3 **The straight driving speed and lane position measures were not significantly different from those during the distracted straight driving conditions**.

**Table:**

**Pairwise comparisons**			
**MEASURE: MEASURE_1**			
**(I) Speed condition**	**(J) Speed condition**	**95% Confidence interval for difference^a^**	
		**Lower bound**	**Upper bound**
1	2	−5.056	−0.459
	3	−7.066	−3.569
	4	−6.571	−3.318
2	1	0.459	5.056
	3	−4.686	−0.435
	4	−4.554	0.179
3	1	3.569	7.066
	2	0.435	4.686
	4	−1.489	2.234
4	1	3.318	6.571
	2	−0.179	4.554
	3	−2.234	1.489

**Table:**

Descriptive statistics						
	***N***	**Minimum**	**Maximum**	**Mean**	**Std. deviation**	**Std. error mean**
Straight speed	16	54.37	67.78	58.5669	3.36209	0.84052
Straight distraction speed	16	55.67	63.50	58.6931	2.33586	0.58397
Straight lane position	16	1.91	2.95	2.3519	0.31327	0.07832
Straight distraction position	16	1.82	3.26	2.5069	0.42180	0.10545

**Table:**

**Paired samples test**				
		**Paired differences**		
		**Mean**	**Std. deviation**	**Std. error mean**
Pair 1	Straight speed—straight distraction speed	−0.12625	2.91779	0.72945
Pair 2	Straight lane position—straight distraction position	−0.15500	0.34065	0.08516

**Table:**

**Paired samples test**			
		**Paired differences**	
		**95 Confidence interval of the difference**	
		**Lower**	**Upper**
Pair 1	Straight speed—straight distraction speed	−1.68103	1.42853
Pair 2	Straight lane position—straight distraction position	−0.33652	0.02652

**Table:**

**Paired samples test**				
		***t***	***df***	**Sig. (2-tailed)**
Pair 1	Straight speed—straight distraction speed	−0.173	15	0.865
Pair 2	Straight lane position—straight distraction position	−1.820	15	0.089

Based on estimated marginal means:

a Adjustment for multiple comparisons: least significant difference (equivalent to no adjustments).
